# The role of mental health on the relationship between food insecurity and immunologic outcome among people living with HIV in Guangxi, China

**DOI:** 10.1080/21642850.2020.1854762

**Published:** 2020-12-08

**Authors:** Cheng Chen, Xueying Yang, Chengbo Zeng, Xiaoming Li, Shan Qiao, Yuejiao Zhou

**Affiliations:** a School of Public Health, School of Medicine, Shanghai Jiao Tong University, Shanghai, People’s Republic of China; b Department of Health Promotion, Education, and Behavior, University of South Carolina Arnold School of Public Health, Columbia, SC, USA; c South Carolina SmartState Center for Healthcare Quality (CHQ), University of South Carolina Arnold School of Public Health, Columbia, SC, USA; d Guangxi Center for Disease Control and Prevention, Nanning, People’s Republic of China

**Keywords:** Mental health, Food insecurity, Immunologic outcome, CD4, HIV/AIDS

## Abstract

**Background:**

Previous studies showed that food insecurity could adversely affect clinical outcomes of people living with HIV (PLWH). The mental health pathways of such effects are suggested in existing literature, but empirical data are limited in resource-limited settings.

**Methods:**

This cross-sectional study aims to explore the role of depressive symptoms and anxiety on the association between food insecurity and CD4 counts among a sample of 2,987 PLWH in Guangxi, China. Path analysis was used to examine a hypothetical model and delta *z* test was used to assess the indirect effects of food insecurity on CD4 counts through depressive symptoms and anxiety.

**Results:**

The prevalence of food insecurity in this sample was 25.3%, and the median CD4 counts were 318 cells/mm^3^. In correlation analyses, food insecurity was not directly associated with LogCD4 but was associated with depressive symptoms and anxiety. Path analysis indicated a significant indirect effect from food insecurity to LogCD4 through depressive symptoms, but not anxiety.

**Conclusion:**

Improving mental health among PLWH with food insecurity may be beneficial for treatment outcomes. Besides intervening food insecurity, an intervention targeting depressive symptoms could improve the immunologic outcomes of PLWH.

## Introduction

According to Chinese Centers for Disease Control and Prevention (CDC), the number of people living with HIV (PLWH) has reached 1.25 million in 2018 (Chinese Centers for Disease Prevention and Control, 2018). With the worldwide availability of antiretroviral therapy (ART), the mortality of PLWH has been decreased in recent years (Herbst et al., 2009; Hogg et al., 1997; Palella et al., 1998), resulting in the longer life expectancy (Antiretroviral Therapy Cohort Collaboration, 2017). However, according to a 2016 meta-analysis, only around 40% of PLWH were on ART and 36% of those on ART achieved viral suppression in China (Levi et al., 2016). While effective ART can significantly improve the immune recovery of the majority of PLWH, there is still a certain proportion of PLWH who remain at relatively low CD4 levels (Kaufmann et al., 2003).

CD4 cells (also known as CD4+ T cells) are white blood cells that fight infection. CD4 count is an indicator of immune function in PLWH and one of the key determinants for the need of opportunistic infection prophylaxis. CD4 counts are obtained from bloodwork as part of laboratory monitoring for HIV infection. As an immunological outcome, CD4 count is a critical indicator for classifying clinical stages of HIV infection, evaluating treatment efficacy, and changing the medication when necessary (Bentwich, 2005). Lower CD4 counts are reported to be associated with an increased risk of opportunistic infection (Lewden et al., 2007). Yet the immunological outcome was not satisfactory in China, with the median CD4 count ranging from only 118 to 182 cells/mm^3^ (Huang et al., 2015; Ruan et al., 2010; Zhang et al., 2009). Therefore, it is important to explore potential factors that impede immunological recovery among PLWH. Factors associated with lower CD4 count in previous studies included older age (Kaufmann et al., 2003), treatment interruption, non-adherence to ART (Gross, Bilker, Friedman, & Strom, 2001; Kaufmann et al., 2011), and food insecurity (Aibibula et al., 2016).

Food insecurity, defined as a limited or uncertain ability to acquire acceptable foods in socially acceptable ways, or limited or uncertain availability of nutritionally adequate and safe foods (Anderson, 1990), has been found to be associated with multiple health problems of PLWH, such as unprotected sex, suboptimal treatment adherence (Weiser et al., 2009b), incomplete viral suppression (McMahon, Wanke, Elliott, Skinner, & Tang, 1999), and greater mortality (Weiser et al., 2009a). Food insecurity is common among PLWH in resource-poor settings, ranging from 24% to 81% (Mamlin et al., 2009; Palar et al., 2015; Wang et al., 2011).

The underlying mechanisms about the negative effect of food insecurity on HIV outcomes can be explained in several perspectives. In pharmacokinetic perspective, food intake is recommended for processing, absorption, and optimal clinical benefits for certain ART regimens. For example, pharmacokinetic studies showed as much as a 30% increase in the plasma concentration of darunavir (Prezista) when taken with food (Gribble et al., 2000) and administration of atazanavir (Reyataz) with a light meal increases drug plasma concentration by 70% (Gribble et al., 2000). Prescribing ART that requires food to those who are food insecure could therefore diminish the efficacy of ART even when patients are adherent (Kalichman et al., 2015). In behavioral perspective, food insecurity can interfere with medication adherence by disrupting daily routines, impairing memory and attention, impeding adherence strategies, and reducing motivation (Frega, Duffy, Rawat, & Grede, 2010 Singer, Weiser, & McCoy, 2015;). In addition, in the face of limited resources, demands for food may compete with resources needed to procure medicines. The need to make tradeoffs between paying for food and other medical care (including transport costs to clinic) leading to poor retention in medical care (Young, Wheeler, McCoy, & Weiser, 2014), which would influence the treatment outcomes (e.g. CD4 counts).

A 2016 literature review (Aibibula et al., 2016) revealed that only 8 studies investigated the association between food insecurity and immunological outcomes (e.g. CD4 counts). According to this systematic review, PLWH with food insecurity had 1.32 times higher odds of having lower CD4 counts compared to PLWH without food insecurity. All 8 studies except one were conducted in the U.S. Thus, there is a dearth of such data in China, such as Guangxi. According to China National Health Accounts Report, Guangxi has a heavy burden on medical expense in 2008 (China National Health Economics, 2005). Guangxi’s poverty rate is 17.1%, compared to the whole China’s 8.3% (Deng, 2012), and the average monthly salary of Guangxi is 1087 yuan, only half of the national average (Gong, 2011). However, there is a paucity of data investigating food insecurity among PLWH in Guangxi.

Given the negative impact of food insecurity on HIV treatment outcomes (e.g. CD4 counts), understanding the underlying mechanisms could potentially inform the future interventions to improve HIV treatment outcomes. Previous literature suggested that inadequate dietary intake can lead to mental health problems (McIntyre, Williams, Lavorato, & Patten, 2013), which in turn leads to compromised immunity among PLWH, especially decrease in CD4 counts (Ickovics et al., 2001; Kemeny et al., 1994; Leserman, 2008; Patterson et al., 1996). Among all the mental health problems, psychiatric problems such as depressive and anxiety symptoms have been reported to be highly prevalent among PLWH (with the prevalence of 36% and 16%, respectively) (Atkinson et al., 2009; Bing et al., 2001 Kemeny et al., 1994;). Moreover, some studies (Burack et al., 1993; Leserman et al., 1997) demonstrated a faster decline in CD4 counts in PLWH with depressive symptom than those PLWH without depressive symptoms. Since food insecurity can interplay with depressive and anxiety symptoms as suggested in the existing literature (Laraia, Siega-Riz, Gundersen, & Dole, 2006; Vozoris & Tarasuk, 2003; Whitaker, Phillips, & Orzol, 2006), it is plausible that these two mental health problems may play a role in the relationship between food insecurity and CD4 counts. Hence, the current study aims to examine the relationship between food insecurity and CD4 counts and to explore the effects of depressive and anxiety symptoms on such relationship in Guangxi, China. We hypothesized that (1) food insecurity is negatively associated with CD4 counts; and (2) food insecurity can affect CD4 counts through depressive symptoms and anxiety.

## Materials and methods

### Study site and participants

Data were derived from a cross-sectional survey conducted in Guangxi, China from October 2012 to August 2013. Details of the survey were described previously (Yang et al., 2019). Briefly, 2 cities and 10 rural counties that had the largest cumulative number of reported HIV/AIDS cases were selected as the survey sites. Staff from local CDC and healthcare workers from local health centers were selected and trained to facilitate the data collection. Individuals who were aged ≥18 years old and with a confirmed HIV diagnosis were eligible to participate. The aforementioned staff conducted face-to-face interviews in private rooms after obtained informed consent from the participants. The latest CD4 counts were retrieved from the participants’ medical records with appropriate informed consent. With an approximate 10% refusal rate, 3002 PLWH were recruited and 2987 completed questionnaires. The study protocol was approved by the Institutional Review Boards at the Wayne State University in the United States and Guangxi CDC in China.

## Measures

### Sociodemographic characteristics

The sociodemographic characteristics included gender, age, ethnicity (Han/others), marital status (single/separated, married/cohabitated, divorced/widowed), religion, education attainment (illiteracy/primary school, middle school, college), employment status (no job, part-time, full-time), place of household registration, and monthly household income (in Chinese currency, RMB). Participants were also asked to provide HIV-related information, including duration since HIV diagnosis and their HIV treatment status. The log transformed CD4 count (LogCD4) was used as the dependent outcome since its distribution was highly skewed (Table 1).

**Table 1.** Socio-demographic characteristics.

		LogCD4	
Variables	*N* (%)	Mean ± SD	*P*
**Total**	2987 (100%)	-	-
**LogCD4**	2855 (95.6%)	5.57 ± 0.93	
**Age** (years)			**<.001**
≤30	404 (13.5)	5.66 ± 1.04	
30-50	1864 (62.4)	5.58 ± 0.94	
>50	719 (24.1)	5.47 ± 0.81	
**Duration of Diagnosis** (Mean ± SD, years)		3.63 ± 2.44	**<.001**
≤5	2230 (74.7)	5.49 ± 0.97	
6∼10	710 (23.8)	5.78 ± 0.75	
>11	47 (1.5)	5.80 ± 0.71	
**Gender**			
Male	1876 (62.8)	5.48 ± 0.96	**<.001**
Female	1111 (37.2)	5.71 ± 0.84	
**Ethnicity**			
Han	2109 (70.7)	5.56 ± 0.94	0.817
Others	873 (29.3)	5.58 ± 0.90	
**Levels of Education**			
Less than primary school	901 (30.3)	5.52 ± 0.91	**0.018**
Middle school or above	2075 (69.7)	5.58 ± 0.93	
**Employment status**			
No job	800 (26.9)	5.45 ± 1.09	**0.001**
Part-time	992 (33.4)	5.57 ± 0.87	
Full-time	1182 (39.7)	5.64 ± 0.83	
**Household’s monthly income (RMB)**			
<2000	2442 (82.6)	5.57 ± 0.92	0.595
≥2000	516 (17.4)	5.56 ± 0.94	
**Marital status**			
Single/Separated	386 (13.2)	5.43 ± 1.10	**0.017**
Married/cohabitated	2011 (68.9)	5.59 ± 0.92	
Divorced/Widowed	520 (17.8)	5.55 ± 0.81	
**Household registration**			
The local city	2783(93.4)	5.57 ± 0.91	0.167
Other cities in Guangxi	168 (5.6)	5.39 ± 1.17	
Other provinces	29 (1.0)	5.79 ± 0.69	
**Whether on ART**			
Yes	2146 (72.1)	5.53 ± 0.93	**<.001**
No	829 (27.9)	5.63 ± 1.05	

Note: * The number of the participants is not equal to the total sample size of some variables due to missing data.Bold values indicate the significant variables with p-value <0.05.

### Food insecurity

Food insecurity was defined by one question: ‘In the past year, does your household have enough food to meet the needs of the whole family? (Yes/no)’. Participants who responded ‘No’ were categorized as experienced food insecurity, while participants who answered ‘Yes’ were categorized as not experienced food insecurity.

### Mental health

Depression: The 10-item Center for Epidemiologic Studies Depression Scale (CESD-10) (Andresen, Malmgren, Carter, & Patrick, 1994), a shortened version from the standard 20-item scale, was used to measure depression (Radloff, 1977). The CESD-10 consists of questions about how many depressive symptoms a person has experienced in the past week (item samples: ‘I can’t concentrate on what I’m doing’, ‘I’m full of hope for the future’, ‘I don’t sleep well’). Each item has four choices from ‘never or seldom’ (Chinese Centers for Disease Prevention and Control, 2018) to ‘often’ (Herbst et al., 2009), resulting in a total score ranging from 10 to 40, with a higher total score representing a higher level of depressive symptoms. The Cronbach alpha for the CESD-10 was 0.79 in this study after reversing certain items.

Anxiety: The Brief Anxiety Scale (Zung, 1971) was used to measure anxiety. The scale consists of 20 questions about the feelings a person has experienced in the past week (item samples: ‘I feel easily upset or panic’, ‘I have a headache and a sore neck’, ‘My face was hot and red’). (Response categories: 1 = never or seldom, 4 = often). A summary score was created by adding up the score of each item, with a higher total score representing a higher level of anxiety symptoms. After reversing certain items, this scale exhibited good internal reliability (Cronbach alpha = 0.90).

### Data analysis

First, descriptive statistics were reported on socio-demographic characteristics (e.g. age, gender, marital status). Mean and standard deviation (SD) were used to describe continuous variables, and frequencies were used for categorical variables. Bivariate analyses were carried out using t tests or ANOVA to examine the relationships between socio-demographic characteristics and logCD4. Second, bivariate correlation analyses were performed to examine the associations among food insecurity, depressive symptom, anxiety, and logCD4. Third, path model was conducted to examine the associations among depressive symptoms, anxiety, logCD4, and food insecurity while adjusting for covariates that were significantly associated with logCD4 in bivariate analyses. Depressive symptoms, anxiety, and logCD4 were standardized before conducting path analysis. Delta *z* tests were used to examine the roles of depressive symptoms and anxiety between food insecurity and logCD4. Both unstandardized (see Appendix) and standardized path coefficients were reported in this study. Descriptive statistics and correlation analyses were performed using SPSS software version 22 (SPSS Inc., Chicago, IL). Path analysis was performed using Mplus version 7.0 (Muthen & Muthen, Los Angeles, CA).

## Results

### Sociodemographic characteristics

As shown in Table 1, among a total of 2987 participants, the average age was 42.46 years old (SD = 12.84); more than half of them (62.8%, 1876/2987) were male. The majority of the participants were of Han ethnicity (70.7%, 2109/2987). Around 70% of participants were married or in a cohabiting relationship (68.9%, 2011/2987), and had received middle school or above education (69.7%, 2075/2987). Most participants (82.6%, 2442/2987) had a household’s monthly income less than 2000 RMB. In terms of the treatment, the majority of them (72.1%) were on ART. The mean scores of CESD-10 scale and anxiety scale were 19.5 (SD = 4.8) and 31.1 (SD = 8.9), respectively.

### Association between background characteristics and outcome

The prevalence of food insecurity among PLWH in this study was 25.3%. The median CD4 count was 318 cells/mm^3^ (interquartile range: 195–459 cells/mm^3^), with the mean and SD of logCD4 of 5.57 (SD = 0.93). Table 1 shows the associations between socio-demographic characteristics and logCD4. PLWH who were female (*p* < .001), younger (*p* < .001), having a full time job (*p* = 0.001), being married/cohabited with boyfriend or girlfriend (*p* = 0.017), and having a household registration of other provinces (*p* = 0.167) were more likely to have a higher logCD4 than their counterparts.

### Correlations among depressive symptom, anxiety, logCD4 and food insecurity

Table 2 shows correlations among depressive symptom, anxiety, logCD4, and food insecurity. Results of correlation analyses indicated that both depressive symptom and anxiety were positively associated with food insecurity, and their Spearman correlation coefficients were 0.242 (*p*< .001) and 0.257 (*p*< .001), respectively. Both depressive symptom and anxiety were negatively associated with logCD4, and their Spearman correlation coefficients were −0.092 (*p*< .001) and −0.080 (*p*< .001), respectively. Food insecurity was not significantly associated with logCD4 (correlation coefficient = 0.003, *p* = 0.89).

**Table 2.** Correlation matrix.

	1	2	3	4
1. Depression	1.000			
2. Anxiety	0.735***	1.000		
3. LogCD4	−0.092***	−0.080***	1.000	
4. Food insecurity	0.242***	0.257***	0.003	1.000

Notes: * *p* < 0.05; ** *p* < 0.01; *** *p* < 0.001.

### Path analysis

While adjusting for covariates that were significantly associated with CD4 counts in bivariate analyses, path model revealed that food insecurity was not significantly associated with logCD4 but was significantly related to both depressive symptom (std.β = 0.229, *p* < .001) and anxiety (std.β = 0.247, *p* < .001). Depression was negatively associated with logCD4 (std.β = −0.062, *p* = 0.041), while the relationship between logCD4 and anxiety was not statistically significant (std.β = −0.004, *p* = 0.897) (Table 3).

**Table 3.** Path coefficients.

Paths*	Std.β	95% C.I.	S.E.	*p*-value
Food insecurity --» Depression	0.229	0.191∼0.268	0.020	**<.001**
Food insecurity --» Anxiety	0.247	0.207∼0.283	0.019	**<.001**
Food insecurity --» LogCD4	0.004	−0.036∼0.042	0.020	0.837
Depression --» LogCD4	−0.062	−0.123∼−0.007	0.041	**0.041**
Anxiety --» LogCD4	−0.004	−0.063∼0.054	0.030	0.897

*Adjusted for significant socio-demographic characteristics in bivariate analyses: age, gender, levels of education, employment status, marital status, whether on ART, and duration of diagnosis.

Bold value indicate the significant associations with p-value <0.05.

### Direct and indirect effects of path model

**Figure 1.**
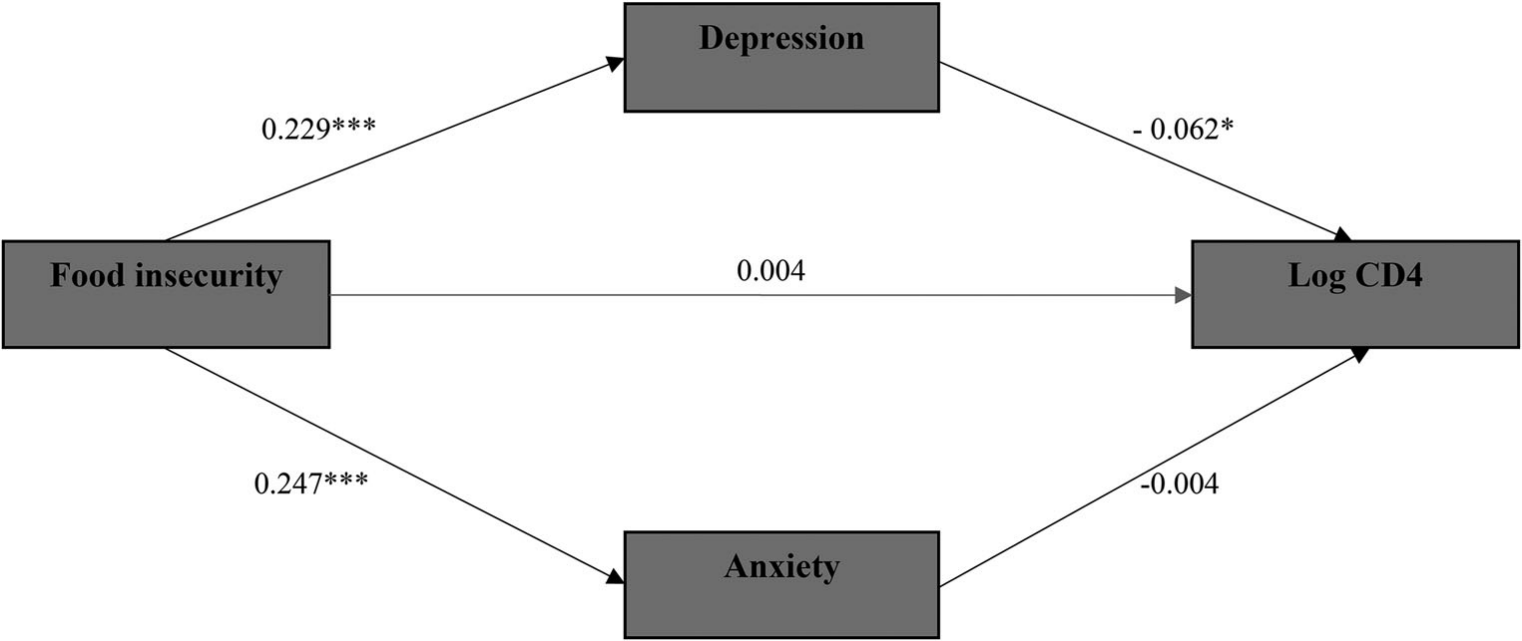
The mediation effects of mental health problems on the relationship between food insecurity and immunologic outcome (i.e. CD4 count). Notes: Covariates (age, gender, employment status, marital status, household registration and whether on ART) with *p*-values less than 0.05 in bivariate analysis were adjusted in the final model. *p < 0.05, **p < 0.01, ***p < 0.001. All path coefficients were standardized coefficients.

Direct pathway from food insecurity to logCD4 was not significant (std.β = 0.004, *p* = 0.837), but indirect pathway from food insecurity to logCD4 through depressive symptom was significant (std.β = −0.014, delta *z* = −2.01, *p* = 0.045) (Table 4). Of note, such indirect pathway through anxiety was not statistically significant (std.β = −0.001, delta *z* = −0.13, *p* = 0.897). This result indicated that food insecurity may affect CD4 count indirectly through depressive symptom but not anxiety (Figure 1). Unstandardized path coefficients were shown in the Appendix.

**Table 4.** Mediation analysis.

Effect*	Std.β	95% C.I.	S.E.	*p*-value
Total effect	−0.011	−0.049∼0.024	0.019	0.550
Indirect effect				**0.002**
Depression	−0.014	−0.028∼−0.001	0.007	**0.045**
Anxiety	−0.001	−0.016∼0.014	0.008	0.897
Direct effect	0.004	−0.036∼0.042	0.020	0.837

*Adjusted for significant socio-demographic characteristics in bivariate analyses: age, gender, levels of education, employment status, marital status, whether on ART, and duration of diagnosis.

Bold value indicate the significant associations with p-value <0.05.

## Discussion

To the best of our knowledge, this is one of the first studies to investigate the relationship between food insecurity and CD4 counts among PLWH in China. The finding underscored food insecurity issue among PLWH in China. Although the prevalence of food insecurity among PLWH in the current study is lower than the rate in other cities of China (e.g. 37.2% in Chengdu) or other countries (e.g. 63% in the US) (Hu et al., 2011; McMahon et al., 1999), the results addressed its role in compromising the immunological recovery of HIV patients in China. Addressing food insecurity should become an integral part of HIV care.

Although no direct effect between food insecurity and CD4 counts was found, results from the current study revealed the indirect association between the two variables through depressive symptom after controlling for potential confounders. Beside intervening food insecurity among PLWH, the government needs to pay more attention to this impoverished population with mental health problems in order to improve their treatment outcomes. These findings highlighted the importance of addressing food insecurity as part of comprehensive HIV care in order to improve HIV-related treatment outcomes.

It’s worth noting that the insignificant association between food insecurity and CD4 counts was not congruent with previous results from existing studies which suggested a negative effect of food insecurity on CD4 counts (Kalichman et al., 2010 Palar et al., 2015; Weiser et al., 2014;). The study settings may partially account for such inconsistency. Compared with China, some previous studies were conducted in countries where the starvation problem is more acute, such as Africa (Benzekri et al., 2017 Weiser et al., 2014;). HIV patients there can be greatly affected by their nutritious status. In addition, different measures of food insecurity could also account for such inconsistency. For example, previous studies measured food insecurity using the Household Food Insecurity Access Scale (HFIAS) (Coates, Swindale, & Bilinsky, 2007) or the US Food Security Scale (Cook & Frank, 2008), but we only used one item and asked participants whether their food is enough for the whole family. Further studies are warranted to use more comprehensive measure to capture food insecurity and confirm the findings of this study. However, the results in this study could still point pinning the food insecurity issues and provide some insights for the future intervention programs.

In this study, food insecurity affected CD4 counts indirectly through depressive symptoms. Based on an established conceptual framework (Weiser et al., 2011), the impacts of food insecurity on clinical outcomes may be explained by behavioral pathways (delayed entry into care, poor clinic attendance, interruptions in care), nutritional pathways (macronutrient and micronutrient deficiencies, worse absorption of drugs in the absence of food), and mental health pathways. The results of this study contributed more evidence pertinent to the mental health pathways in that conceptual framework. Food insecurity is a source of significant distress and shame (Mickelson & Williams, 2008). Studies have documented associations between food insecurity and depressive symptoms in HIV population (Palar et al., 2015; Vogenthaler et al., 2011). Previous studies even have found the dose–response relationship between food insecurity and depressive symptoms among women living with HIV (Palar et al., 2018). Depression in common among PLWH and is associated with declining CD4 counts in previous studies (Olisah, Adekeye, & Sheikh, 2014). The negative effects of depression on immune status may consequently result in HIV disease progression. The WHO recommends that attention to psychosocial needs of PLWH should be an integral part of HIV care (World Health Organization, 2005). PLWH need extra emotional care and psychological support to prevent and cope with mental health problems in order to promote immunological recovery. Further investigation of this pathway is needed in larger studies including detailed measures of macronutrient and micronutrient deficiencies. Future interventions should pay more attention on PLWH at a risk of food insecurity with depression or anxiety problems to improve their immune system.

There are some limitations to be aware of in the current study. First, the one-item measure of food insecurity may not accurately capture the practical situation of food insufficiency among PLWH. Second, this is a cross-sectional study, the casual relationships cannot be drawn from this study. Further studies should estimate causality between food insecurity and CD4 counts employing longitudinal study designs. Third, self-report bias, including recall bias and social desirability bias may exist during the data collection. Thus, the rate of food insecurity in this study might be underestimated. Fourth, the effect size of the associations in this study was small. There might be some other covariates or confounders that mediate or moderate the relationships we did not consider. We called for future research to investigate the mediators or moderators in the relationships between psychosocial variables and immunologic outcome. Finally, our results are based on a sample of HIV patients in Guangxi, China. It is necessary to be cautious when extending these findings to other settings.

Despite these limitations, the findings in this study revealed the food insecurity issues among PLWH in China and suggested that food insecurity might compromise the immunologic outcome of HIV patients through depressive symptom. This link sheds light on the importance of addressing food insecurity as part of comprehensive care to improve health outcomes among PLWH. Improving mental health among PLWH with food insecurity may be beneficial on treatment outcome. Future research is needed to better understand the underlying mechanisms and explore other potential pathways.

## Author contributions

Conceptualization, X.Y.Y. and X.M.L.; methodology, X.Y.Y. and C.B.Z.; software, C.B.Z.; validation, X.M.L.; formal analysis, C.C. and C.B.Z.; writing-original draft preparation, C.C.; writing-review & editing, X.Y.Y.; project administration, S.Q. All authors have read and agreed to the published version of the manuscript.

## Supplementary Material

Supplemental Material
