# Supplementary material for: The role of mental health on the relationship between food insecurity and immunologic outcome among people living with HIV in Guangxi, China
Source: Health Psychol Behav Med. 2020 Dec 8;8(1):636–48. doi: 10.1080/21642850.2020.1854762 (PMC7993086; doi:10.1080/21642850.2020.1854762)
Supplement: Supplemental Material [file RHPB_A_1854762_SM9103.docx]

Appendix 1 Path coefficients with non-standardized β results

| Paths* | β | 95% C.I. | S.E. | *p*-value |
| --- | --- | --- | --- | --- |
| Food insecurity --» Depression | 2.561 | 2.120~3.015 | 0.226 | **<.001** |
| Food insecurity --» Anxiety | 5.059 | 4.206~5.888 | 0.430 | **<.001** |
| Food insecurity --» LogCD4 | 0.009 | -0.079~0.093 | 0.043 | 0.837 |
| Depression --» LogCD4 | -0.012 | -0.025~-0.001 | 0.006 | **0.042** |
| Anxiety --» LogCD4 | 0.000 | -0.007~0.006 | 0.003 | 0.897 |

*Adjusted for significant socio-demographic characteristics in bivariate analyses: age, gender, levels of education, employment status, marital status, whether on ART, and duration of diagnosis.

Appendix 2 Mediation analysis with non-standardized β results

| Effect* | β | 95% C.I. | S.E. | *p*-value |
| --- | --- | --- | --- | --- |
| Total effect | -0.025 | -0.108~0.054 | 0.042 | 0.549 |
| Indirect effect | -0.034 | -0.056~-0.013 | 0.011 | **0.002** |
| Depression | -0.032 | -0.064~-0.002 | 0.016 | **0.045** |
| Anxiety | -0.002 | -0.036~0.030 | 0.017 | 0.897 |
| Direct effect | 0.009 | -0.079~0.093 | 0.043 | 0.837 |

*Adjusted for significant socio-demographic characteristics in bivariate analyses: age, gender, levels of education, employment status, marital status, whether on ART, and duration of diagnosis.
